# Sustainable practice change: Professionals' experiences with a multisectoral child health promotion programme in Sweden

**DOI:** 10.1186/1472-6963-11-61

**Published:** 2011-03-22

**Authors:** Kristina Edvardsson, Rickard Garvare, Anneli Ivarsson, Eva Eurenius, Ingrid Mogren, Monica E Nyström

**Affiliations:** 1 Department of Public Health and Clinical Medicine, Epidemiology and Global Health, Umeå University, SE 901 87 Umeå, Sweden; 2 Division of Quality Management, Luleå University of Technology, SE 971 87 Luleå, Sweden; 3 Department of Clinical Science, Obstetrics, Umeå University, SE 901 87 Umeå, Sweden; 4 Medical Management Centre, Department of Learning, Informatics, Management and Ethics, Karolinska Institutet, SE 171 77 Stockholm, Sweden

## Abstract

**Background:**

New methods for prevention and health promotion and are constantly evolving; however, positive outcomes will only emerge if these methods are fully adopted and sustainable in practice. To date, limited attention has been given to sustainability of health promotion efforts. This study aimed to explore facilitators, barriers, and requirements for sustainability as experienced by professionals two years after finalizing the development and implementation of a multisectoral child health promotion programme in Sweden (the Salut programme). Initiated in 2005, the programme uses a '*Salutogenesis*' approach to support health-promoting activities in health care, social services, and schools.

**Methods:**

All professionals involved in the Salut Programme's pilot areas were interviewed between May and September 2009, approximately two years after the intervention package was established and implemented. Participants (n = 23) were midwives, child health nurses, dental hygienists/dental nurses, and pre-school teachers. Transcribed data underwent qualitative content analysis to illuminate perceived facilitators, barriers, and requirements for programme sustainability.

**Results:**

The programme was described as sustainable at most sites, except in child health care. The perception of facilitators, barriers, and requirements were largely shared across sectors. Facilitators included being actively involved in intervention development and small-scale testing, personal values corresponding to programme intentions, regular meetings, working close with collaborators, using manuals and a clear programme branding. Existing or potential barriers included insufficient managerial involvement and support and perceived constraints regarding time and resources. In dental health care, barriers also included conflicting incentives for performance. Many facilitators and barriers identified by participants also reflected their perceptions of more general and forthcoming requirements for programme sustainability.

**Conclusions:**

These results contribute to the knowledge of processes involved in achieving sustainability in health promotion initiatives. Facilitating factors include involving front-line professionals in intervention development and using small scale testing; however, the success of a programme requires paying attention to the role of managerial support and an overall supportive system. In summary, these results emphasise the importance for both practitioners and researchers to pay attention to parallel processes at different levels in multidisciplinary improvement efforts intended to ensure sustainable practice change.

## Background

Vast evidence shows that conditions during the foetal period, infancy, and childhood can affect physical and mental health throughout life [1-4]. Although chains of risk factors for physical and mental problems can be interrupted by preventive and health promoting interventions [5], current research shows that the rate of adoption, implementation, and sustainability of such interventions often is low, indicating that many potential health benefits are never achieved [6-11]. For example, a recent Swedish child health care intervention project in Uppsala County aimed to broaden the psychosocial support to families; however, the intervention resulted in only a few families taking part in the originally planned interventions, and professionals were more likely to distribute books and brochures instead of changing their working routines [12].

Precisely why changes do or do not occur in multifaceted preventive programs can be difficult to explain [13]. A number of factors are important - independently or in interaction with others [14] - and barriers that may impede change of perceptions, attitudes, and behaviours among professionals can be found at different levels of health care [15,16]. To improve quality and outcomes of care, one needs to take into account factors specific to the levels of the individual, group or team, organization, and the larger environment [15].

Implementation research deals with questions such as "what is happening and why"? [17], and theories on implementation of change can be used to explain under what circumstances change most likely will be achieved [18]. Sustainability is a key to programme success and can be defined as 'the degree to which an innovation continues to be used after initial efforts to secure adoption is completed' [19]. However, it is well known that compliance rates often drop and return to pre-intervention levels when specific implementation efforts have ended [6,20], and one question still remains unanswered: What are the crucial components that lead to sustainability of innovations in health care [21,22]? Quantitative studies have dominated this field of research, but more qualitative studies are needed [14]. Qualitative methods can further the understanding of why or why not sustainability can be reached, for example, by exploring reasons behind certain behaviours among professionals [23]. To contribute to a deeper understanding of these processes, we explored facilitators, barriers, and requirements for programme sustainability as experienced by involved professionals two years after finalizing the development and implementation of a multisectoral child health promotion programme in Sweden.

## Methods

### Study context

The study was conducted in Västerbotten County, Sweden (260,000 residents). In 2005, Västerbotten County Council launched the Salut Programme - a multisectoral child health programme developed to support the provision of health promoting activities in health care, social services, and school settings. The programme has a '*Salutogenesis*' approach, which implies focusing on factors that support human health and well-being rather than factors that cause disease [24]. Starting with the pregnant woman and her partner, the programme continues to follow the child, partly by involving parents, up to 18 years of age through age specific modules. The programme also includes an epidemiological surveillance component. This study covers the first two modules that target parents and their children from foetal life to 1½ years of age.

### Description of involved sectors

In Sweden, nearly all health care is provided through a national social insurance system, mainly financed through taxes levied by county councils and municipalities [25]. The maternal and child health services, which are part of this system, are free and reach nearly all expectant women and children aged 0 - 6 years in the country.

Antenatal care with registered midwives responsible for activities provides women with counselling and interventions regarding sexual and reproductive health and maternal and foetal surveillance during pregnancy. Pregnant women are offered seven to nine visits from the first trimester to childbirth, additional counselling by physicians if required, and a follow-up visit 6-12 weeks post partum [26]. Child health care staffed by registered nurses with qualification in child health provide families with support, advice, and information regarding issues such as child health and development, immunization, breast feeding, nutrition, child safety, and parenting. Visits to child health care centres are recommended at approximately 11 key ages during the child's first 18 months and subsequently at 3, 4, and 5 years of age. Examination by physicians are included in five of these visits [27].

In Sweden, dental care can be provided by the Public Dental Services or by private care providers. The County Councils responsibility is to ensure that dental care is available to everyone and free comprehensive dental care is provided for children up to the age of 19 [28]. Open pre-schools offer pedagogical group activities led by preschool teachers and serve as alternatives to the regular pre-school for children with parents on parental leave or non-working. These services are free, children are not registered, and they are not obliged to attend regularly [29].

### Salut programme development and implementation process

**Figure 1:**
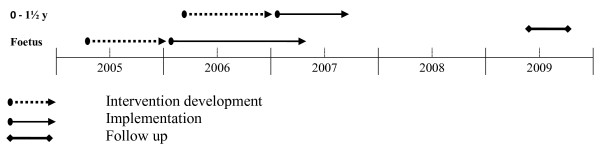
**Timeline for intervention development, implementation, and follow up on sustainability of modules targeting parents and their children from foetal life to 1½ years of age**.

Health-promoting interventions targeting children (foetal life to 1½ years of age) and their parents were developed and implemented in each sector in the four pilot areas between 2005 and 2007. A modified version of the Institute for Health Care Improvement's Breakthrough Series Model guided the intervention development process [30], supported by the County Council change process consultants and led by the Salut Programme management. Professionals in the pilot areas attended five learning seminars and conducted small-scale testing of interventions between seminars for one year, guided by the principles of the Plan-Do-Study-Act (PDSA) cycle of learning [31]. Then the intervention package was decided upon jointly by the professionals, Salut Programme managers, and experts in the field of maternal, child, and dental health. The year of intervention development was followed by a one-year implementation period that provided another five learning seminars to help the participants improve their skills, adjust their interventions, and evaluate the feasibility of the programme. All seminars during the first and second year included lectures on topics related to relevant health issues and discussions in small groups on the progress of the programme using the following questions: What has happened since the last seminar? How do we proceed? What is our plan for small-scale testing? The seminars also provided the participants with tools such as manuals and some practical training. Two outreach visits to each group were performed by the Salut programme managers during the intervention development and implementation periods. The 'salutogenetic' approach was not new to the Swedish health care system, as preventive measures such as counselling on healthy life habits previously had been part of the service in most sectors investigated. The majority of the participants had a short education in 'Motivational interviewing' [32]. However, further development of the professional's knowledge and skills were facilitated by the combination of lectures, group discussions, and small-scale testing of interventions. The resulting intervention package, which was summarized in work manuals and included structured protocols and questionnaires, is presented in Table 1. The timeline for intervention development, implementation, and follow up on sustainability of modules targeting parents and their children from foetal life to 1½ years of age is presented in Figure 1.

**Table 1:** The intervention package within the Salut Programme targeting parents and their children from foetal life to 1½ years of age

Intervention	Antenatal care	Child health care	Dental service	Open pre-school
Motivational interviewing [32]	*	*	*	
Collaboration between involved sectors	*	*	**	**
Parent meetings	*	*	**	**
Health counselling focusing on life habits, mental health, domestic violence^1^, parent-child attachment, psychosocial health and parent relationships	*	*	**	
Edinburgh Postnatal Depression Scale (EPDS) screening [61]		*		
Oral health screening at 12 months of age		**		
"Mothers visit" at child age 8 months including screening for domestic violence		**		
"Fathers visit" at child age 10 months with focus on fathers experiences of change in life situation		**		
Questionnaires for health surveillance	**	**	**	
Free dental health care visit for the pregnant woman and her partner			**	
Activities to enhance early parent-child attachment, children's physical activity and linguistic development		*		*
Activities supporting parents to establish contacts with each other				*
Activities to promote healthy snacks/food and drinks				*

* Strengthening or restructuring of existing interventions

** Newly developed interventions within the Salut Programme

^1 ^Pregnant women and women recently given birth

### Participants

All of the professionals (n = 23) involved in the pilot areas of the Salut Programme gave their consent to participate in the study after an invitation via telephone by the first author. Hence, the study included the whole eligible population. Participants' characteristics are presented in Table 2.

**Table 2:** Characteristics of the participants in the study (n = 23)

	Sex female, n (%)	Age mean, years (range)	Work experience mean, years (range)
Midwives	5 (100)	53 (41-64)	24 (16-32)
Child health nurses	7 (100)	57 (52-63)	26 (13-38)
Dental hygienists/dental nurses	7 (100)	39 (26-47)	14 (05-29)
Pre-school teachers	4 (100)	54 (48-58)	31 (26-35)

### Data collection procedures

Semi-structured face-to-face interviews [23] were conducted at each working site from May to September 2009 - approximately two years after that the intervention package was established and the implementation phase had ended. Two participants requested to be interviewed simultaneously; all others were interviewed individually. The interviews lasted between 25 and 55 minutes (mean 33 minutes). Participants were asked to describe and reflect on the following experiences: i) participating in the development process of the programme; ii) the current situation in their work place in relation to programme activities; iii) facilitators and barriers for compliance to the programme; iv) general views on important requirements for continuous development and programme sustainability; and v) other thoughts or reflections in relation to these themes that they wanted to include. All interviews were digitally recorded.

### Data analysis

Verbatim-transcribed data underwent qualitative content analysis through a systematic classification process, and coding into categories provided information on the latent and manifest content [33,34]. First, the interviews were read several times to get a holistic sense of the content. By this, the individual participants' perceived sustainability of the programme also became known. Second, data was coded to capture key thoughts and concepts related to facilitators, barriers, and requirements for sustainability. Third, codes with shared conceptual content were sorted into broad content areas and subsequently abstracted into categories. Fourth, the content of all categories were validated against the verbatim-transcribed data. Finally, a model inspired by Grol and Wensing was used to sort categories into a theoretical scheme [16]. This multi-level model proposes factors to be identified at the levels of the innovation, the individual professional, the patient, the social context, the organizational context, and the economic and political context. The software Open Code 3.4 was used as a tool for coding and categorizing all data [35]. In the result section, we use the following concepts to describe proportions of participants contributing to a specific category: *Few *refer to 1-4, *some *to 5-9, *half *to 10-14, *most *to 15-19, and *all *to 20-23 participants. Quotations are provided to illustrate how the interpretations are grounded in data.

### Trustworthiness

The first author conducted the interviews and completed the primary analysis and developed codes and preliminary categories, which then were reviewed against the original interview transcripts by two co-authors independently. To strengthen the credibility and dependability of the analysis, several interviews were also independently read by the other researchers [33]. The authors were largely in agreement about the conceptualization. Nevertheless, during the course of analysis, uncertainties in coding and interpretation were regularly and thoroughly discussed by all authors to reach consistent findings. The first author, who is a registered nurse with work experience in child health care was familiar with the study context but did not occupy dual roles. The co-authors' various backgrounds - paediatrics, epidemiology, public health, work and organizational psychology, engineering and quality management, physiotherapy, and obstetrics and gynaecology - provided complementing perspectives that enriched the analysis process and interpretation of the results.

### Ethics approval

All participation was based on informed consent. Ethics approval was obtained from the Regional Ethical Review Board in Umeå, Sweden (08-168Ö).

## Results

### Perceived programme sustainability

The programme was described as sustainable at most sites with the exception of child health care where few participants reported complete or high sustainability of the programme. The following two quotations are examples of how participants described high versus low level of programme sustainability.

*This way of working is so well established so I know what to do.... I don't have to read the manuals frequently*. (Dental hygienist)

*It [the programme] does not work; for me it's not working at all right now. I feel that I'm back in my old routines because that's the easiest and fastest way*. (Child health nurse)

Several factors of importance for programme sustainability were identified during analysis. Table 3 gives an overview of perceived facilitators, barriers, and requirements nested in the theoretical scheme inspired by Grol et al. [16] with one level added, the development process. Main findings at each of the included levels are summarized below.

**Table 3:** Factors influencing the Salut Programme sustainability, nested in a theoretical scheme inspired by Grol and Wensing [16]*

	FACTORS INFLUENCING SUSTAINABILITY		
**LEVEL**	**REQUIREMENTS**	**FACILITATORS**	**BARRIERS**
			
**Innovation**			
Development process		Involvement in development and small scale testing ^1-4^ Support from process consultants ^1-4^ Having time to develop strategies ^1-4^	Time consuming and ineffective ^1-4^

Content	Carefully designed work manuals ^3^	Perceived as important ^1-4^ Easily integrated ^1-4^ Manuals essential tools ^1-4^ Clear programme branding ^1-4^	Time consuming ^1-4^ Not suiting specific needs of immigrants ^1,2,4^ Difficulty with social and psychological problems ^1,2^ Found similar to approaches already present at the work place ^1,2,4^

**Individual**			
Professionals	Own commitment and interest ^1-4^	Own values coherent with programme's purpose and goals ^1-4^	Lack of motivation ^2,3,4^ Programme goals found unrealistic ^2^
Parents (Patients)		Positive attitudes to interventions ^3,4^	New topics and questionnaires intrusive and extensive ^1-3^ Content of parent meetings unpopular ^1,3^

**Context**			
Social	Regular meetings ^1-4^ Permanent programme organization ^1-3^ Information to new employees ^1,3^ Managerial responsibility and commitment^1-4^	Regular meetings ^1-4^ Active managerial support ^1-4^	Lack of managerial involvement or support ^1-4^ Lack of involvement or support from physicians or other colleagues ^1-3^

Organizational	Programme integrated in action plans ^1-3^ Geographical proximity for collaborators ^1-4^ Sufficient time ^1-3^ Further establishment and spread of the programme ^1-4^	Geographical proximity for collaborators ^1-4^	Lack of time and resources ^1-4^ Lack of communication and agreement between programme management and local managers ^2,3^

Economical and political	Incentives in line with programme intentions ^3^		Conflicting incentives for performance ^3^ Threat of cutbacks ^1-3^

^1 ^Represent the views of midwives, ^2 ^child health nurses, ^3 ^dental hygienists/dental nurses, ^4 ^open pre-school teachers

* With an added level; development process.

### Perceived facilitators, barriers, and requirements for programme sustainability

#### The innovation development process

During the analysis, several facilitators related to the process when the interventions were developed and tested discerned. Most participants experienced learning seminars followed by small-scale testing to be an efficient way to translate sweeping visions and challenging goals to small and feasible interventions. This facilitated programme sustainability and an overall understanding of the programme.

*At first, we had enormously high goals set that were unrealistic. They have been adjusted into smaller goals by us. I think, that is why we are here today*. (Dental hygienist)

However, some participants experienced the involvement in the development process as demanding and highly time consuming, especially since they also felt that they did not move forward. This led to lowered motivation.

*We were so tired of all those questions, about current status and how to proceed. At the same time we felt that we did not move forward, we were stuck at square one*. (Open pre-school teacher)

The County Council change process consultants' support was seen as highly valuable in the development process as it facilitated structured and goal-oriented work and feedback on performance. Some participants described how their motivation increased as a result of being given power to influence the development process and programme content. A few stated that the programme was their 'own' product, something that they claimed had facilitated sustainability.

*We have built this on our own. It had empty spaces, lacked a basic programme, had nothing like this. It is ours, definitely ours*. (Open pre-school teacher)

The importance of being given time to practice new ways of working and thus speed up the learning curve during the start-up period was experienced as facilitating programme sustainability by half of the participants.

*If you can learn to do a good job, then I think that will lead to success.... if you for a while have time to develop a good routine, then I think it will be sustainable*. (Child health nurse)

*It does not take much more time if you have time to practice and introduce it as a part of your working methods*. (Midwife)

#### The innovation content

All participants described the programme's relevance for promoting the health of expectant parents, children, and their parents as an important facilitator, although a few expressed a decrease of motivation and interest in the programme since components of the intervention were perceived as being similar to approaches that were already present at the workplace. The content of the intervention was seen as being up to date, enhancing the ability of viewing the family as a unit, and in line with values, working methods, and goals of the participants. This relevance was described as facilitating the integration of programme activities in daily work.

*It fits my way of thinking. In that way it has been easy*. (Child health nurse)

Most of the participants viewed the manuals, including protocols and questionnaires, as facilitating discussions on sensitive topics and as a key to a standardized way of working. They were also seen as an important requirement for programme sustainability, for example, by serving as support during periods of staff turnover.

*Well-documented work manuals are important; it is essential that new employees, regardless of the place and profession, easily can get information on how we work*. (Dental hygienist)

One concern shared by most participants was that using the established intervention package was time consuming. The comprehensive interventions in child health care (Table 1) were experienced as a major barrier for sustainability, as these required extra time from an already resource-constrained sector. Some participants also emphasised that the programme was not sufficiently tailored to meet the needs of immigrants. A few mentioned psychosocial aspects as difficult or challenging to deal with, such as defining good psychosocial health, raising questions about it, or handling existing problems.

*There are a lot of things that comes up to the surface. The hard thing is to know how to deal with it in a good way*. (Midwife)

Some participants mentioned that parents often recognized the Salut Programme brand in different settings after the initial introduction in antenatal care; this exposure helped them recognize what the programme represented. One nurse described how she experienced Salut as self-selling, since parents were asking to also involve their residential area in the programme. The brand name was also experienced as a facilitator for carrying out the interventions, for example, when professionals referred to the programme when raising uncomfortable questions. It also meant that professionals could identify themselves as being part of a team and a larger effort.

*You just have to mention Salut when you call, then everybody knows why you are calling. Because everyone has heard of it*. (Dental hygienist)

#### The individual professionals

Half of the participants expressed that their personal values corresponded to the programme's purpose and goals and experienced this as a strongly supporting factor for integrating and continuously carrying out the Salut activities, while a few experienced these goals as unrealistic. Some participants mentioned that being committed and interested in the programme were important requirements for programme sustainability. However, a few participants noted barriers related to lack of motivation that, for example, was a result of not being able to participate at meetings and thus 'losing the thread' of the discussion, or just being tired of the recurring introductions of new working methods.

*I cannot say that it has been difficult... it has not been like that, but... I mean everything that is new. If you've been working as long as I have, you sometimes feel that, oh no, please, no more.... Do you understand? You know, something more to be put on your shoulders*. (Child health nurse)

Another barrier was experiencing competing health messages in other contexts, resulting in perceptions of interventions as redundant.

#### The Parents (Patients)

Some participants stated that parents that were positive towards and embraced the interventions facilitated programme sustainability, while some experienced problems with parents that perceived the new topics of questionnaires and discussions as extensive and intrusive. A few also stated that the new topics developed within the Salut programme for parents' meetings were not popular among parents. This sometimes led to cancellation of meetings.

*The clients experiences that there are too many questionnaires. Many questions and forms, they obviously get tired of it, which is understandable*. (Dental hygienist)

#### The social context

Regular meetings with involved professionals from different sectors within the Salut programme were by most seen as strongly valuable and stimulating. Continuous learning was facilitated by sharing knowledge, advice, and ideas, and by giving and getting feedback. The meetings also facilitated insight in the different professions' activities and ideas on how synergies could be created by collaboration. Half of the participants stated that regular meetings were a crucial component for the programme's survival; a few specifically mentioned the need of a permanent programme organization to support continuing networking activities.

*When the programme is disseminated, I believe that coordinators are needed in all areas.... you really have to have some unifying persons, otherwise it will disappear*. (Dental hygienist)

*It is always like, it is always a lot of enthusiasm in the beginning of a project, and then, you will fall back into old routines. I think it's necessary to stop and think, and come together in meetings and things like that*. (Midwife)

*I thought it was fantastic to be there, and to benefit from other's knowledge, to learn new things I can use in my work. And, of course, I share my knowledge as well, and in that way I am part of the decision process*. (Pre-school teacher)

Lack of support and involvement from managers as well as lack of formal mandate to drive change were by half of the participants experienced as existing or potential barriers. Both active and passive managerial support was discussed. Participants who described a high level of programme sustainability also experienced active leadership. However, some participants described managers who expressed the importance of the programme, but did not actively support its progress and the professionals themselves felt full responsibility for programme continuation. For example, some participants had to ask for sanctions to carry out programme activities and managers failed to give priority to the programme and had to be 'reminded' to ensure the basic conditions for the interventions. A few experienced their managers to be dissatisfied with the programme since they thought it was too time consuming and did not give enough in return to the clinic. Managerial commitment and responsibility were declared as important requirements for sustainability. A few participants also emphasized the need to integrate the programme with organizational action plans.

*It is important to have support from our leaders.... I think it is important to remind them in some way, for example to go to their meetings and to remind them now and then in between. I think that is important to keep the flame burning*. (Midwife).

*The managers have a great responsibility in leading [leadership]. If they stop talking Salut, then I think Salut will die, actually, I think that's the fact*. (Dental hygienist)

*The managers have not been present. They have not given the priority to this programme as they perhaps should have done, partly perhaps or because they relied on the project coordinator. They saw her as the spider of the web and the one who should spread the information. So they could withdraw themselves a bit*. (Open pre-school teacher)

Another barrier, experienced by half of the participants, was the difficulty finding support among colleagues and/or involvement from physicians within the work place or support from colleagues outside the pilot areas.

#### The organizational context

Geographical proximity (i.e., working in the same building or in the same neighbourhood) was by most participants experienced as a strong facilitator, but also as a requirement for multisectoral collaboration, one of the programme's cornerstones. Synergies were created by spontaneous contact or interaction when sharing premises.

If you have a query it is so easy just to walk over there [to collaborators in other sectors] because we are so close. It's essential that it is easy. (Dental hygienist)

The sub-group of participants reporting low compliance to the programme described the organizational level barriers as the main reasons for not carrying out the Salut interventions, with lack of time and resources frequently mentioned as barriers. Time restraints resulted not only in prioritizing 'ordinary' tasks prior to tasks related to the Salut programme, but also hindered the participants from finding their own strategies to incorporate the programme activities into daily work. A few also expressed that the lack of communication and agreement between the Salut programme management and local managers resulted in a gap between resources and the programmes' intention.

*This extra work has been forced into our regular activities and working hours, and that is never good. You need extra time during the start up period in order to find your own solutions.... You basically need time to develop this. And this extra time was never given us*. (Child health nurse)

Further establishment and spread of the programme were by some seen as an important requirement for sustainability; one participant characteristically questioned if it was worth the effort working with the programme if not all areas in the county would be involved in the near future.

#### The economic and political context

Barriers at this level largely concerned dental health care, where professionals felt the pressure of generating money from their clinics by treating adults, while in the Salut programme expectant parents were offered a free visit. Even though most participants from dental care claimed high compliance with the programme two years after its implementation, these conflicts between different incentives were described as a threat for long-term sustainability.

We are doing this now because we think it's fun and interesting. However, it will be difficult to involve others, because it is more important to have patients that generate money. This generates no money. (Dental hygienist)

Threats of cutbacks were also experienced by some as reducing motivation and leading to prioritizing other tasks before programme interventions.

*This [The Salut programme] is unfortunately nothing that is given priority right now because of the threats of cutbacks.... These things are given the lowest priority under such circumstances, that's the way it is*. (Child health nurse)

## Discussion

To our knowledge, this is the first study that explores factors of importance for sustainability of a multisectoral child health promotion programme in a Swedish context. The programme was described as sustainable at most sites, except in child health care. The perception of facilitators, barriers, and requirements were largely shared across sectors. Facilitators included being actively involved in intervention development and small-scale testing, personal values corresponding to programme intentions, regular meetings, working close with collaborators, using manuals, and a clear programme branding. Existing or potential barriers included insufficient managerial involvement and support and perceived constraints regarding time and resources. In dental health care, barriers also included conflicting incentives for performance. Many facilitators and barriers identified by participants also reflected their perceptions of more general and forthcoming requirements for programme sustainability. From our point of view, this strengthens the importance of these factors.

The theoretical framework proposed by Grol and Wensing [16] was found to be feasible in structuring results of this study, findings that support its usefulness as a multilevel approach to examine factors of importance for sustainability of innovations. This framework has been used in previous studies to identify facilitators and barriers for change [36,37]. However, our results contribute to extend the framework by also including the level of the development process, as several facilitating factors were found at this level. Other theoretical frameworks (for example, as proposed by Cabana et al.) might also have been applicable [38].

Professionals' participation early in the process of programme development and the use of small-scale testing were described as strongly contributing to programme sustainability. During that process, interventions became context adapted and a sense of ownership of the programme was fostered on behalf of the professionals. These results are consistent with previous research findings regarding positive aspects of involving front-line professionals in intervention development [19,39-41]. The risk of low awareness and limited practical use of guidelines that mainly were developed at managerial levels has previously been recognized [42].

The difficulties of sustaining long-term compliance rates are well known [6,20]. Therefore, the relatively high level of perceived programme sustainability among professionals in this study is an interesting finding, especially since well-recognized barriers in terms of insufficient support from managers and peers as well as understaffing and time constraints were reported from all sectors [43]. These factors mainly affected professionals in child health care, and lack of sustainability in this sector might be attributable to a more comprehensive intervention package and a more pressed work situation. The relatively high age of the involved child health nurses could be a contributing factor, as older and experienced professionals tend to use guidelines to a lesser extent compared to their younger and less experienced peers [43].

Most participants viewed the use of manuals, including protocols and questionnaires, as highly valuable in achieving sustainability by facilitating a standardized way of working and by serving as supporting tools when raising sensitive questions. The use of growth charts has previously shown to facilitate raising issues about overweight in child health care [44], and structured protocols for screening has shown to raise awareness and improve documentation of child abuse among emergency department staff [45]. However, previous qualitative research regarding the role of manuals as tools in similar health promotion initiatives is scarce and further studies are needed.

The perceived attributes of the innovation, including relative advantage, compatibility, complexity, trialability, and observability can, according to Rogers, explain between 49% to 87% of the variance in the rate of adoption [19]. Furthermore, interventions that can easily be tried out in practice and that do not need additional resources are more likely to be implemented [43]. Professionals' training has also been found to be a crucial factor in achieving sustainability of health education programmes in schools [46]. These factors were also facilitated because of the chosen strategy to involve front-line professionals in programme development.

However, there are conflicting messages concerning whether guidelines developed by involved professionals themselves are used more often or not [43]. There is also criticism of the 'participation model' concerning the risk of not introducing the best care possible and the risk of not paying attention to structural factors that are important for successful implementation [20]. Some factors were found to serve as both facilitators and barriers for sustainability. One example is the experience of being motivated by involvement, but at the same time facing lack of managerial support when given authority to participate in programme development. This phenomenon has previously been examined [47,48]. In this study, the advantages of the participation model were challenged by the perceived difficulties at the organisational level. Results indicate that there might be risks for less programme sustainability if managerial levels are not involved and if an organizational structure for continuing support and development is not sustained [49,50]. Furthermore, conflicting incentives for performance, as described by professionals in dental health care, might also pose a threat to long-term programme sustainability; clearly, these conflicts should be taken into account. Hence, this study highlights the importance of planning for sustainability at an early stage in programme development [51] and of analysing both target contexts and target groups before intervention and implementation designs are set [52-54].

Multidisciplinary collaboration is often aimed for in health services, but it is rarely achieved with ease. Our results indicate that teamwork can be enhanced and synergies created by regular meetings and by sharing premises or having geographical proximity. This might be important to consider for those who have mandate to decide on the organization of services for expectant parents and young children that depend on multisectoral collaboration. These results also appear consistent with previous results regarding facilitators and barriers to such collaboration [55]. Stability in the work force from programme initiation until the time when the study was undertaken likely contributed to programme sustainability. Otherwise, high rates of professionals' turnover can undermine existing collaboration as key persons leave their positions [56,57].

The role of the programme's brand name in facilitating for professionals to raise uncomfortable questions with clients was a somewhat unexpected finding. Thus, not only is a brand name important in relation to the adoption of health behaviour of individuals [58]. The right branding might also serve as a facilitator for clarifying the programme's mission and goal, encouraging behavioural change among professionals working in the field of health promotion. Interestingly, this seems to be an often overlooked factor in previous discussions of facilitating factors for behavioural change among this diversity of professionals, even though the importance of communicating visions and goals has been recognised.

### Methodological discussion

A qualitative approach with inductive coding and categorizing [34,53] was considered appropriate since studies of barriers and facilitators in similar contexts as well as implementation studies involving other professionals than physicians are sparse [48]. Rich data were obtained as all professionals in the pilot areas consented to take part in the study [59,60]. The importance of having an open-ended approach was confirmed since an existing framework used for organizing findings was expanded. In addition, the similarity of issues raised by participants regardless of profession indicates that our findings might also be transferable to other professions and settings. As the study covered the whole eligible population, we decided to specify some quantities in the results section. However, the quantification of data should be interpreted with caution, and our results cannot be considered as exhausting the area of barriers and facilitators due to their sensitivity to the intervention, target group, and context. A limitation of this study is that it reflects the views of the programme's front-line professionals. Perspectives of people at the managerial levels and of the receivers of interventions (i.e., expectant parents and parents) would add value and provide a more comprehensive picture of important factors of sustainability. A more objective assessment of sustainability is also warranted. Furthermore, the study reflects the perceptions of female participants of similar age. Nevertheless, the proportion of women in this study mirrors the general female predominance in these sectors in Sweden. A re-organization into family centres during the start-up phase of the Salut Programme might have influenced our results as it led to closer proximity and opportunities for collaboration between maternal health care, child health care, open pre-school, and social services. Due to this, it might be possible that the importance of having collaborators nearby were raised to a greater extent. Finally, because of the recent launch of the programme interventions, their effectiveness has not yet been evaluated or reported, something that otherwise would have strengthened this study.

## Conclusions

These results contribute to the knowledge of processes involved in achieving sustainability in health promotion initiatives. Facilitating factors include involving front-line professionals in intervention development and using small-scale testing; however, the success of a programme clearly requires paying attention to the role of managerial support and an overall supportive system. In summary, these results emphasise the importance for both practitioners and researchers to pay attention to parallel processes at different levels in multidisciplinary improvement efforts intended to ensure sustainable practice change.

## Competing interests

The authors declare that they have no competing interests.

## Authors' contributions

KE, MN, RG, and AI designed the study. KE performed the interviews, conducted the initial analysis, and wrote the manuscript. All authors reviewed, edited, and approved the final manuscript.

## Pre-publication history

The pre-publication history for this paper can be accessed here:

http://www.biomedcentral.com/1472-6963/11/61/prepub
